# Notes and News

**Published:** 1897-05-15

**Authors:** 


### NOTES AND NEWS.

(Collected and Communicated.)

The Prince of Wales has fixed Wednesday, May 26th, as the date on which he will open the new Medical School buildings at Guy's Hospital.

The Queen has forwarded her usual subscription of £105 to the funds of the Middlesex Hospital, through Sir Fleetwood Edwards.

Dr. Charles Knott, of Southsea, has sent £200 to the treasurer of Guy's Hospital, being the balance of the £500 raised in Portsmouth for the endowment in perpetuity of a "Tiny Tim Cot" in memory of Charles Dickens.

The Duke of Fife, K.T., will take the chair at the forty-fifth annual general court of governors of the Hospital for Sick Children, Great Ormond Street, to be held at the hospital on Wednesday, May 26th, at half-past four p.m.

We regret to announce the death of Mr. Walter Rivington, consulting surgeon to the London Hospital, from influenza. He was a member of the council of the Royal College of Surgeons and of the senate of the London University.

The Duke and Duchess of Connaught will lay the foundation-stone of the new wing of the Royal Portsmouth Hospital in August. The scheme will cost £14,000. The foundation-stone of the original building was laid by the Prince Consort 50 years ago.

The Duke of Cambridge, Vice-President of the Middlesex Hospital, will preside at the quarterly court of governors of that institution which has been specially convened for Thursday, 27th inst., at noon, for the purpose of passing an address to the Queen on the occasion of Her Majesty's Diamond Jubilee.

Mr. W. Youngman has made a most generous offer to the governors of the Lowestoft Hospital. He proposes to build and furnish a children's wing to the present building, and the suggestion has been gratefully accepted. An appeal will be made to the public for funds for endowment, to provide additional funds and increased subscriptions for its maintenance.

The Staffordshire General Infirmary has just been reopened after very extensive structural alterations and improvements. A considerable portion of the old building has been pulled down and new wings erected, the central block being remodelled for administrative purposes. The formal reopening ceremony took place in the board-room on April 21st, when the buildings were declared open by the Countess of Lichfield, in the presence of a large gathering. The Bishop of Lichfield was present, the Earl of Lichfield, the Earl and Countess of Harrowby, the Earl and Countess of Dartmouth, and the High Sheriff (Mr. W. B. Harrison).

